# Phosphoinositide-3-kinases p110α and p110β mediate S phase entry in astroglial cells in the marginal zone of rat neocortex

**DOI:** 10.3389/fncel.2013.00024

**Published:** 2013-03-14

**Authors:** Rabea Müller, Catharina Fischer, Thomas Wilmes, Bernd Heimrich, Vanessa Distel, Norbert Klugbauer, Dieter K. Meyer

**Affiliations:** ^1^Institute of Experimental and Clinical Pharmacology und Toxicology, Albert-Ludwigs-University FreiburgFreiburg, Germany; ^2^Institute of Neuroanatomy, Albert-Ludwigs-University FreiburgFreiburg, Germany

**Keywords:** bromodeoxyuridine, glycogen synthase kinase-3β, p27^Kip1^, marginal zone, p110α, p110β, rat neocortex

## Abstract

In cells cultured from neocortex of newborn rats, phosphoinositide-3-kinases of class I regulate the DNA synthesis in a subgroup of astroglial cells. We have studied the location of these cells as well as the kinase isoforms which facilitate the S phase entry. Using dominant negative (dn) isoforms as well as selective pharmacological inhibitors we quantified S phase entry by nuclear labeling with bromodeoxyuridine (BrdU). Only in astroglial cells harvested from the marginal zone (MZ) of the neocortex inhibition of phosphoinositide-3-kinases reduced the nuclear labeling with BrdU, indicating that neocortical astroglial cells differ in the regulation of proliferation. The two kinase isoforms p110α and p110β were essential for S phase entry. p110α diminished the level of the p27^Kip1^ which inactivates the complex of cyclin E and CDK2 necessary for entry into the S phase. p110β phosphorylated and inhibited glycogen synthase kinase-3β which can prevent S-phase entry. Taken together, both isoforms mediated S phase in a subgroup of neocortical astroglial cells and acted via distinct pathways.

## Introduction

Class I phosphoinositide-3-kinases (PI3-kinases) generate phosphoinositide(3,4,5)P_3_ [PtdIns(3,4,5)P_3_] by phosphorylating phosphoinositide(4,5)P_2_ (Wymann and Pirola, 1998; Cantley, 2002). The kinases are heterodimers of a regulatory subunit and a catalytic subunit, such as p110α, p110β, p110γ, or p110δ. Apart from their functions in cell metabolism, survival and migration, the PI3-kinases play important roles in the regulation of the cell cycle in normal and cancer cells (Kuemmerle and Bushman, 1998; Cantley, 2002; Zhao et al., 2003; Wymann and Marone, 2005). During the G0 phase, they can mediate the renewed entry into the cell cycle. They can also facilitate the progression through the G1 phase of the cell cycle as well as the G2/M transition (Garcia et al., 2006; Marques et al., 2008).

Initiated by MAP kinases, the protein complexes cyclin E/CDK2 and cyclin D/CDK4/6 cause the sustained hyperphosphorylation of the retinoblastoma protein and thus initiate S phase entry and DNA synthesis (Garcia et al., 2006; Cross et al., 2011). PI3-kinases can facilitate the formation and activity of cyclin/CDK complexes. In aortic smooth muscle cells and mouse embryonic fibroblasts, PI3-kinases suppress the activity of p27^Kip1^ (Bacqueville et al., 1998; Collado et al., 2000), which inhibits the cyclin E/cdk2 complex (Chu et al., 2008). In addition, PI3-kinases phosphorylate glycogen synthase kinase-3β (GSK-3β) at Ser9. This inactivates the GSK-3β which can prevent S phase entry (Cross et al., 1995; Diehl et al., 1998; Liang and Slingerland, 2003; Yang et al., 2006; Mao et al., 2009).

In a pilot study with cultured rat neocortical astroglial cells, the PI3-kinase inhibitor wortmannin (Arcaro and Wymann, 1993) reduced by approximately 40% the number of cells which showed nuclear uptake of bromodeoxyuridine (BrdU), suggesting that PI3-kinases regulated S phase entry in a subgroup of glial cells. In the present study, we investigated which p110 isoforms were involved in this effect. After identifying the isoforms expressed in astroglial cells, we inactivated them by expressing dominant negative (dn) isoforms in the cells or by using pharmacological inhibitors. Our results show that p110α suppressed the activity of p27^Kip1^, whereas p110β phosphorylated GSK-3β. We also studied the neocortical location of the cells which were regulated by PI3-kinases. Both PI3-kinase isoforms facilitated S phase entry only in astroglial cells located in the marginal zone (MZ) of the neocortex.

## Materials and methods

### Antibodies, drugs, and plasmids

The following antibodies were used: anti-phospho-Akt (Ser473), anti-Akt, anti-p27^Kip1^, anti-phospho-GSK-3β (Ser9), anti-GSK-3β 27C10 (Cell Signaling, Danvers, MA); anti-GAPDH (Millipore GmbH, Billerica, MA), anti-BrdU (Roche Diagnostics GmbH, Mannheim, Germany). Peroxidase-coupled secondary antibodies were bought from Rockland/Biomol GmbH (Hamburg). The following drugs were used: wortmannin, MG-132 (Sigma-Aldrich, St. Louis, MO), AS-252424, TGX-221 (Axxora, Lörrach, Germany); CHIR-99021 (Axon Medchem, Groningen, NL). dn p110β^K805R^, wildtype (wt) p110β, and dnp110α^K833R^ were kindly provided by Nürnberg (Tübingen, Germany); dnp110α^K802R^ and wtp110α were gifts of Wyman (University Basel, Switzerland). Oligonucleotides for PCR analysis were bought from Biomers (Ulm, Germany).

### Primary cultures of dissociated neocortical astroglial cells

Brains from newborn rats were dissected to culture cells from total neocortex. To prepare cultures from the MZ or deep layers of the neocortex, we used brains from 5 day old (P5) rats. The areas were dissected from cooled, 1 mm thick coronal brain slices. As described previously (Hildebrand et al., 1997), cells were dispersed with trypsin (Sigma-Aldrich, München, Germany) and then suspended in Dulbecco's Modified Essential Medium supplemented with 10% endotoxin-free fetal bovine serum (FBS; Biochrom AG, Berlin, Germany). Viable cells were seeded in uncoated six-well plates (35 mm diameter, Greiner Bio-One GmbH, Frickenhausen, Germany). Seeding density was 2.5 × 10^6^ cells per well. The DMEM incubation medium contained 10% FBS and was renewed after 4 days. Experiments were performed with semi-confluent cells after 8–10 days *in vitro* (DIV). Cultures of rat hippocampal neurons were prepared as described previously (Benz et al., 1998).

### Organotypic cultures of neocortex

Brain hemispheres were dissected from neonate rat pups (P0–P2) and cut into 400 μm coronal cortical sections with a McIlwain tissue chopper under sterile conditions. Intact slices were placed on humidified porous Millipore membranes in 6-well plates containing 1.2 ml of serum-based nutrient medium (25% heat-inactivated horse serum, 50% MEM, and 25% Hanks' balanced salt solution supplemented with glutamax at 2 mM final concentration). The medium was changed three times per week.

### Reverse transcription PCR

The RNA of astrocytes cultured from total neocortex and rat spleen tissue was isolated with the RNeasy Mini Kit (Qiagen, Hilden, Germany) according to the manufacturer's protocol. One μg of RNA was transcribed into cDNA with Superscript II Reverse Transcriptase (Invitrogen, Karlsruhe, Germany). For PCR amplification the following primers were used: p110α (forward): 5′-TGCGGCCGCCAGGTAGAGGCCATGGAGAA-3′, p110α (reverse): 5′-TCCATGGGATCTCATTGTTCTGAAACA-3′; p110β (forward): 5′-AGCGGCCGC-AAACAGGTTGAAGCACTCAA-3′, p110β (reverse): 5′-CCCATGGCACCGCGTCCTCT-CCAAAGG-3′; p110γ (forward): 5′-TGCGGCCGCCTGAGAAGTATGATGTCAGT-3′, p110γ (reverse): 5′-CCCATGGCAATGGTTTCATTGGATAGG-3′; p110δ (forward): 5′-TGCGGCCGCGTGCTGATGAAGCAGGGGGA-3′, p110δ (reverse): 5′-CCCATGGG-CCTGCCTTCTCGCTGCTGT-3′. The PCR products were verified by sequence analysis.

### Cell lysis and western blot analysis

Western blot experiments were repeated twice. For total cell extracts, cells were lysed in 1× SDS sample buffer (62.5 mM Tris-HCl, pH 6.8, 2% w/v SDS, 10% glycerol, 50 mM DTT, 0.01% bromophenol blue). Equal amounts of cellular protein lysate were separated on 12.5% polyacrylamide gels and transferred to polyvinylidene difluoride membranes. After treatment with 5% non-fat dry milk for 1 h, membranes were incubated with respective primary antibodies at 4°C overnight and then incubated with an appropriate horseradish peroxidase-conjugated secondary antibody (Biotrend, Köln, Germany). Bound antibodies were detected using enhanced chemiluminescent detection reagent [100 mM Tris HCl, pH 8.0, 1 mM luminol (Fluka/Sigma, Schnelldorf, Germany), 0.2 mM p-coumaric acid, and 3 mM H_2_O_2_], and visualized with the imaging system LAS-3000 (Fujifilm, Düsseldorf, Germany).

### Nuclear uptake of bromodeoxyuridine (BrdU)

BrdU is incorporated into the nascent DNA during the S phase of the cell cycle. Astroglial cells were synchronized by FBS withdrawal for 24 h. If not stated otherwise, 15 h after re-addition of FBS (final concentration 10%), BrdU (10 μM; Roche Diagnostics GmbH, Mannheim, Germany) was added to the medium for the last 60 min of the incubation. Cells were fixed with methanol (−20°C), washed with PBS for 5 min, and then incubated with 2 N HCl (10 min, 37°C). After the medium had been neutralized with borate buffer (0.1 M, pH 8.5), the cells were washed and incubated for 3 h with a mouse anti-BrdU antibody (Roche Diagnostics GmbH, Mannheim, Germany). The immune complex was detected with a Cy3 labeled secondary goat anti-mouse antibody (Dianova, Hamburg, Germany). To estimate the total cell number, fixed cells were stained with 4′, 6-diamidine-2′-phenylindole dihydrochloride (DAPI, Invitrogen, Karlsruhe, Germany) for 10 min.

### Immunocytochemistry in slice cultures

Slice cultures were fixed with 4% PFA in 0.1 M PBS for 2 h. After several washes with PBS, the Millipore membrane with the cultures was cut off, mounted on a planar agar block and re-sliced into 50 μm sections with a vibratome. Denaturation of DNA was achieved by immersion of free floating sections in 2 N HCl at 37°C for 30 min. After several rinses in PBS, slices were incubated in a blocking PBS solution containing 5% normal goat serum (Vector Labs.) and permeabilized with 0.1% Triton-100 for 30 min. For immunolabeling of proliferating astrocytes a polyclonal rabbit anti-GFAP antibody (dilution: 1:500, DAKO) and a mouse anti-BrDU antibody (dilution: 1:1000, DAKO) were used in PBS containing 1% NGS at 4°C overnight. After three washes with PBS for 5 min each, the slices were incubated with secondary antibodies (Cy3-conjugated goat-anti-rabbit IgG, 1:800, Jackson Immuno Research; Alexa 488 goat-anti-mouse IgG, 1:400, Invitrogen) for 2 h at room temperature in the dark. The slices were washed three times in PBS followed by DAPI nuclear stain (1:50,000 Molecular Probes) for a few seconds. Finally, the slices were extensively washed in PBS for 1 h, mounted on glass slides and coverslipped. Slices were digitally photographed (Zeiss ApoTome).

### Infection of astroglial cells with adenovirus systems containing recombinant p110 isoforms

The adenoviral constructs used expressed the respective p110 isoform linked to enhanced green fluorescent protein (EGFP). The non-catalytic p110 mutants still bound to regulatory subunits and thus acted in a dn manner. The following recombinant and wt subunits of PI3-kinase were used: dnp110β^K805R^ and wtp110β (Yart et al., 2002), dnp110α^K802R^ and wtp110α (Wymann et al., 1996) as well as dnp110γ^K833R^ (Ma et al., 1998). The graded expression of dnp110β^K805R^, wtp110β, and dnp110γ^K833R^ was induced with the adenoviral Tet-On system (BD Adeno-X Tet-On Expression System 2; Clontech Laboratories, Mountain View, CA) (Gossen and Bujard, 1992). The respective coding regions were cloned into the tetracycline-responsive pAdeno-X vector. The vector was modified by introducing the coding sequence for EGFP and an internal ribosomal entry site (IRES) into the EcoRI and XhoI sites. Following propagation in HEK 293 cells, the viral lysate was purified by filtration (pore size 0.45 μm). Use of the Adeno-X Rapid Titer Kit (Clontech Laboratories) gave infectious units in the range of 3–7 × 10^9^/ml. All constructs were verified by sequence analysis. Six to seven days after seeding, neocortical astroglial cells were infected for 24 h with recombinant adenovirus and Tet-On virus [in a ratio of 1:1; multiplicity of infection (MOI) ~10]. To induce expression of EGFP and the protein of interest, doxycycline (600 nM) was added to the incubation medium for the subsequent 24 h. After this time, the actual experiments were performed. Cultures were only used, if the infection rate was ≥ 90%, measured by comparing EGFP positive cells to DAPI positive cells.

For the expression of dnp110α^K802R^ and wtp110α the pAdeno-X Expression System 1 (Clontech) was used. The coding regions were cloned into the shuttle vector pShuttle2, which was modified by introducing an IRES-EGFP insert. Then each construct together with the IRES-EGFP insert was cloned into the pAdeno-X vector. Propagation and purification of the viral particles as well as determination of the viral titers were performed as described above. Astroglial cells were infected with recombinant adenovirus 6–7 days after seeding (MOI ~10) and the actual experiments were performed 48 h after viral infection. In all experiments, we obtained an infection rate ≥ 90% measured by comparing EGFP positive cells to DAPI positive cells.

### SiRNA transfection

Semi-confluent astroglial cells were transfected with 100 nM siRNA using DharmaFECT1 (Dharmacon, Lafayette, CO) according to the manufacturer's protocol. The following siRNA-oligonucleotides (sense strand) were used: p27^Kip1^siRNA: GCU CCG AAU UAA GAA UAA U (Qiagen, Hilden; Germany); GSK-3β siRNA: CGA UUA CAC GUC UAG UAU A (Qiagen, Hilden, Germany). A non-silencing RNA consisting of a scrambled sequence (AllStars Negative Control siRNA, Qiagen, Hilden, Germany) was used as a control. Experiments were performed 48 h after siRNA transfection.

### Data collection and statistical evaluation

Experiments were repeated at least once. For measurement of BrdU uptake within one experiment, at least three wells of a six-well plate were used per group. BrdU as well as DAPI positive cells were counted in an area of 0.1 mm^2^ with use of a Zeiss Axiophot microscope to calculate the ratio. Five areas were counted per well. Within one experiment, the mean value of the BrdU/DAPI ratios of the controls was used to express all groups as percent of controls. Results from all experiments were compiled for statistical evaluation, which was made by Kruskal–Wallis' test followed by Mann–Whitney *U*-test. Shown are Means ± CI (0.95%), (*n*) indicates the number of all areas counted.

## Results

### p110α and p110β regulate nuclear BrdU uptake in cultured neocortical astroglial cells

**Figure 1:**
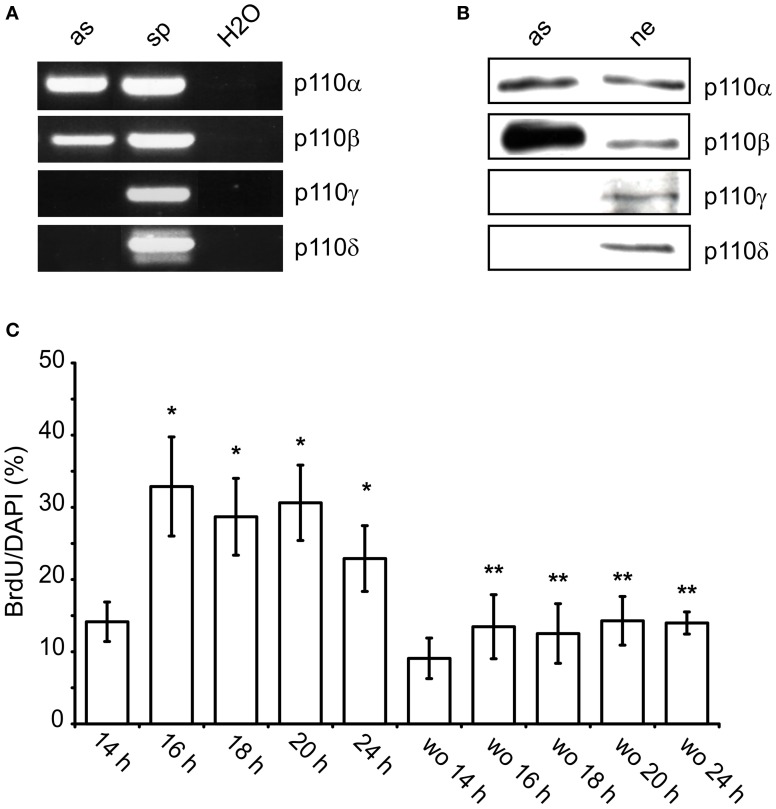
**p110α and p110β are expressed in cultured neocortical astrocytes and facilitate nuclear uptake of BrdU. (A)** RT-PCR for p110α, p110β, p110γ, and p110δ in astroglial cultures (as) and spleen tissue (sp). Samples containing only water (H_2_O) were used as negative controls. **(B)** Western blot from lysates of cultured astroglial cells (as) and hippocampal neurons (ne). **(C)** Time-course of the nuclear uptake of BrdU after re-addition of FBS. To astroglial cells FBS-starved for 24 h 10% FBS^±^ wortmannin (100 nM, wo) was added for the indicated time periods. BrdU was present during the last hour of incubation with FBS. Means ± CI (0.95) are shown; *n* ≥ 15; ^*^~ *p* < 0.05 compared to 14 h, ^**^~ *p* < 0.05 compared to respective cultures without wortmannin.

After 10 DIV, cultures prepared from whole neocortex of newborn rats contain mainly astroglial cells of type I (≥96%) as indicated by the presence of glial fibrillary acidic protein and the absence of A2B5 antigen (Hildebrand et al., 1997). Such cultures were used to characterize the expressed PI3-kinase isoforms. Using RT-PCR and Western-blotting we found only p110α and 110β (Figures 1A,B). In contrast, spleen tissue and cultured hippocampal neurons additionally expressed p110γ or p110δ.

The role of PI3-kinases in S phase entry was studied in semi-confluent cultures which were synchronized by FBS withdrawal for 24 h. Re-addition of FBS increased the number of nuclei which had taken up BrdU. After 14 h, approximately 14% of DAPI stained nuclei were BrdU labeled (Figure 1C). This ratio increased to approximately 30% between 16 and 20 h. It dropped again to 23% after 24 h (Figure 1C). When wortmannin (100 nM) was added together with FBS, it reduced the number of BrdU positive nuclei by 40–60% at all time-points measured (Figure 1C). Apparently, PI3-kinases facilitated G1/S phase transition in a subpopulation of the astroglial cells.

**Figure 2:**
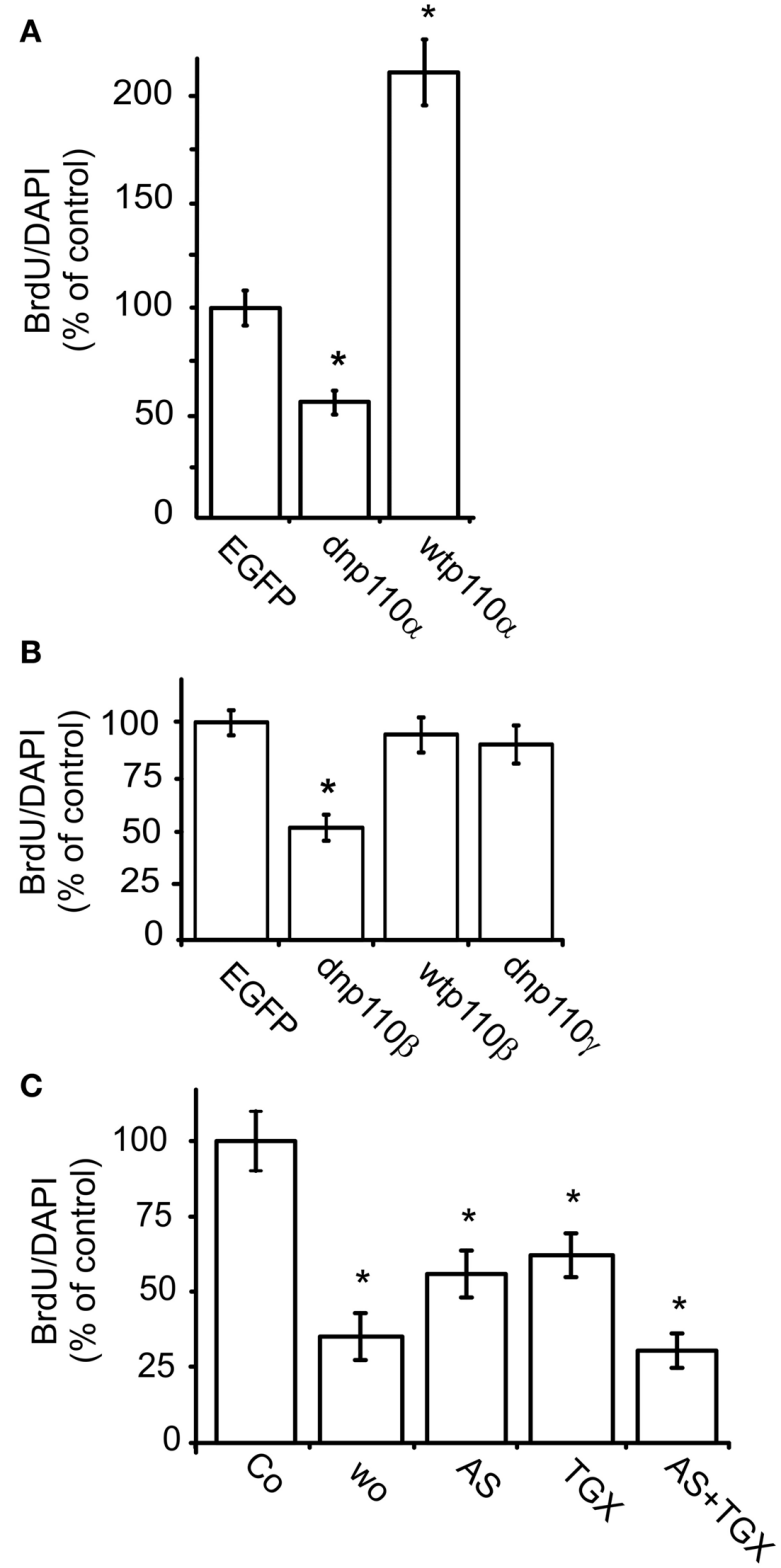
**Inhibition of p110α and p110β reduces the nuclear uptake of BrdU. (A)** Effects of dnp110α^K802R^ (dnp110α) and wtp110α (*n* = 70); **(B)** effects of dnp110β^K805R^ (dnp110β), wtp110β, and dnp110γ^K833R^ (dnp110γ) (*n* values between 40 and 60); **(C)** effects of AS-252424 (5 μM, AS), TGX-221 (200 nM, TGX), and wortmannin (100 nM, Wo) (*n* values of all groups 30). In all experiments, FBS withdrawal for 24 h was followed by re-addition of 10% FBS re-addition for 16 h. Transfections of recombinant p110 isoforms had been performed as described in Materials and Methods. Pharmacological inhibitors were applied together with FBS. Means ± CI (0.95) are shown; ^*^~ *p* < 0.05 compared to control.

Next, we studied the roles of p110α and p110β in nuclear BrdU uptake. To inhibit p110α, we infected the cells with an adenviral construct which caused the expression of dnp110α^K802R^. Since p110γ is not expressed in our cultures, we additionally used the pharmacological agent AS-252424, which inhibits p110α and p110γ (Condliffe et al., 2005). Compared to the respective controls, expression of dnp110α^K802R^ or application of AS-252424 (5 μM) reduced the percentage of BrdU labeled nuclei by 50 and 42%, respectively (Figures 2A,C). In contrast, overexpression of wtp110α increased it by nearly 120% compared to the EGFP controls (Figure 2A).

**Figure 3:**
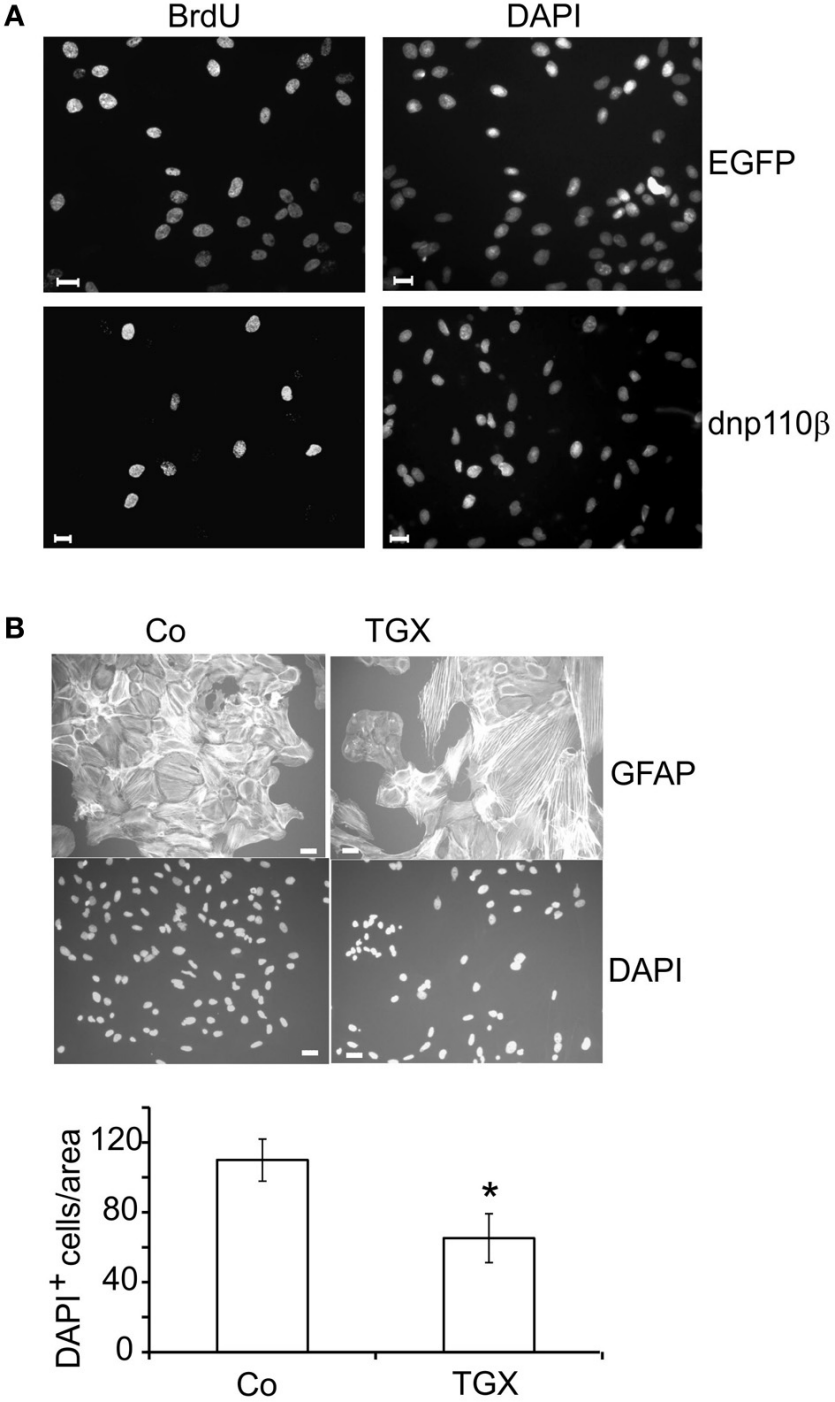
**Short- and long-term effects of 110β inhibition in neocortical astroglial cells. (A)** Transfection with dnp110β (lower panels) decreases nuclear staining of BrdU (left panels). Cells transfected with vector coding for EGFP (upper panels) are shown as controls. DAPI staining shows all nuclei (right panels). **(B)** TGX-221 (200 nM) decreases the total cell number, when applied for 3 days. GFAP staining (upper panels); DAPI staining (lower panels). **(B)** Quantitation of DAPI staining; means ± CI (0.95); ^*^~ *p* < 0.05 compared to controls; *n* ≥ 12; scale bars ~ 10 μm.

To inhibit p110β, we infected the cells with an adenoviral construct which caused the expression of dnp110β^K805R^ or used TGX-221, a pharmacological inhibitor. TGX-221 inhibits p110β 10 times more potently than p110δ (Condliffe et al., 2005), which is not expressed in our cells. The concentration of TGX-221 used in these experiments was too low to affect p110α and p110γ (Condliffe et al., 2005). Expression of wild type p110β did not change the number of BrdU labeled nuclei compared to EGFP controls, but dnp110β^K805R^ reduced it by approximately 50% (Figures 2B, 3A). TGX-221 (200 nM) reduced the number of BrdU labeled nuclei by 38% (Figure 2C). The combination of As252424 and TGX-221 reduced the number of BrdU labeled nuclei stronger than their single application. When combined, they decreased the nuclear BrdU labeling by 60% as did wortmannin (Figure 2C). Dnp110γ^K833R^ did not change the number of BrdU nuclei (Figure 2B). As detailed in Materials and Methods, we used the ratio of BrdU and DAPI stained nuclei, to quantify the effects of the various treatments on DNA synthesis. Therefore, it was necessary to study, whether the treatments changed the number of DAPI stained nuclei. When expressed for 24 h, none of the recombinant PI3-kinases the number of DAPI stained nuclei [means ± CI (0.95) per area]: EGFP controls 64.4 ± 10.7; dnp110α^K802R^ 69 ± 12; wtp110α 60 ± 12.5; dnp110β^K805R^ 55.5 ± 18; wtp110β 55.3 ± 5.5; dnp110γ^K833R^ 71.5 ± 27.9. When present for 16 h, also the pharmacological inhibitors did not affect these numbers [means ± CI (0.95) per area]: control 117 ± 45.3; AS-252424 125 ± 32.3; TGX-221: 118 ± 33.6; wortmannin: 119 ± 54.9. When we applied TGX-221 for 3 days, however, it significantly reduced the number of DAPI stained nuclei by approximately 40% (Figure 3B), indicating a decrease in total cell number. This reduction was in agreement with an inhibitory effect on proliferation.

### p110α facilitates G1/S phase progression by reducing p27^Kip1^ levels

**Figure 4:**
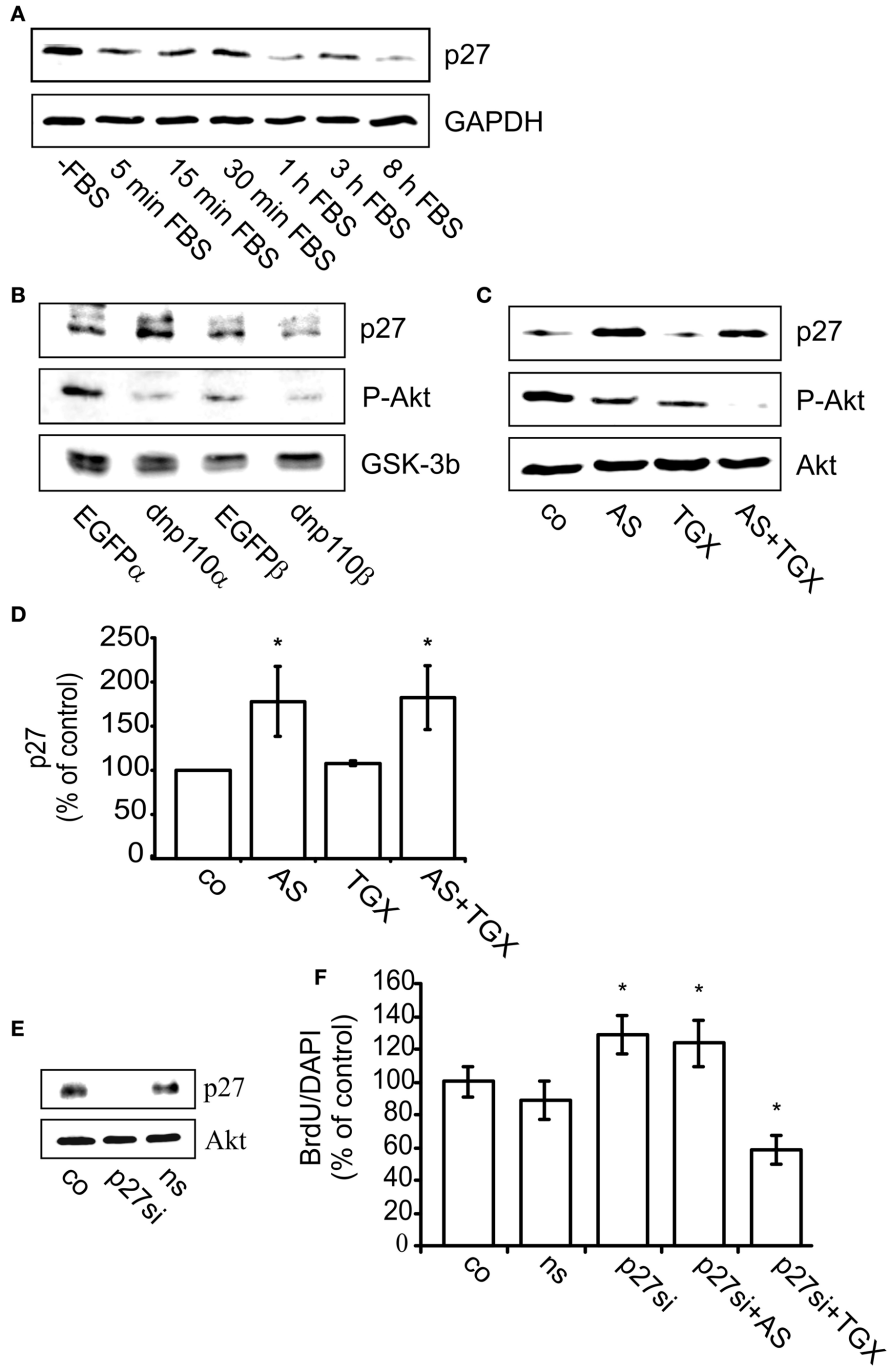
**Inhibition of p110α enhances the cellular level of p27^Kip1^ and thereby reduces BrdU uptake. (A)** Upper lane: p27^Kip1^ levels in cells maintained in FBS-free culture medium (-FBS) for 24 h or after re-addition of 10% FBS for 5 min up to 8 h. Lower lane: GAPDH served as input control. **(B)** Upper lane: levels of p27^Kip1^ in cells expressing dnp110α^K802R^ (dnp110α) or p110β^K805R^ (dnp110β). After prior FBS withdrawal for 24 h, 10% FBS had been added for 8 h. Controls for dnp110α ~ EGFPα; controls for dnp110β ~ EGFPβ ;P-Akt levels are shown the middle lane. GSK-3β served as input control (lower lane). **(C)** Upper lane: levels of p27^Kip1^ in cells treated with AS-252424 (5 μM, AS) and/ or TGX-221 (200 nM, TGX). After prior FBS withdrawal, 10% FBS which contained the drugs had been added for 8 h; P-Akt levels (middle lane); total Akt served as input control (lower lane). **(D)** Quantification of the Western blot experiments. The ratio of the density of the p27 and total Akt bands was calculated; means of controls were taken as 100% [means ± CI (0.95) are shown; *n* = 5; ^*^~ *p* < 0.05 compared to control]. **(E)** Upper lane: levels of p27^Kip1^ protein in cells transfected with p27^Kip1^siRNA (p27si) or non-silencing RNA (ns). Cells were transfected for 48 h. During the last 24 h, they were maintained in FBS-free medium. Total Akt served as input control (lower lane). **(F)** Effects of AS-252424 (5 μM, AS) and TGX-221 (200 nM, TGX) on nuclear BrdU uptake in cells transfected with p27^Kip1^siRNA (p27si); non-silencing RNA (ns). Cells were transfected for 48 h. During the last 24 h they were FBS-starved. Subsequently, 10% FBS was re-added for 16 h. The pharmacological inhibitors were present during this time. The number of BrdU positive nuclei was compared to all nuclei present as stained with DAPI. Means ± CI (0.95) are shown; *n* ≥ 30 for all groups, ^*^~ *p* < 0.05 compared to respective controls.

PI3-kinases diminish the cytosolic and nuclear levels of the CDK-inhibitor p27^Kip1^ and thereby maintain the activity of cyclin E/CDK2 complexes (Bacqueville et al., 1998; Collado et al., 2000; Graff et al., 2000; Vervoorts and Luscher, 2008). When FBS was removed from the incubation medium for 24 h, the cytosolic p27^Kip1^ level was high, whereas re-addition of FBS reduced it by 65% within 5 min. This effect persisted for 8 h, the longest time-point measured (Figure 4A). Eight hours after re-addition of FBS, dnp110α^K802R^ strongly increased the level of p27^Kip1^ compared to the EGFP control. In contrast, expression of dnp110β^K805R^ was ineffective, although dnp110β^K805R^ reduced the phosphorylation of P-Akt as effectively as dnp110α^K802R^ (Figure 4B). Only the p110α inhibitor AS-252424 but not the p110β inhibitor TGX-221 increased the levels of p27^Kip1^ (Figures 4C,D), confirming the results obtained with the dn isoforms.

Inhibition of p27^Kip1^ synthesis with p27^Kip1^siRNA indicated that p27^Kip1^ was indeed relevant for nuclear BrdU uptake. Compared to controls, transfection of the cells with non-silencing RNA neither affected the cytosolic levels of p27^Kip1^ nor the number of BrdU labeled nuclei (Figure 4E). In contrast, transfection of the cells with p27^Kip1^siRNA abolished p27^Kip1^ and increased by 30% the number of nuclei which had taken up BrdU (Figures 4E,F). In such cells, AS-252424 indeed no longer reduced the nuclear uptake of BrdU. In contrast, TGX-221 strongly reduced BrdU uptake in cells transfected with p27^Kip1^siRNA (Figure 4F), confirming that it did not act via p27^Kip1^.

### p110β promotes G1/S phase transition by inactivating GSK-3β

**Figure 5:**
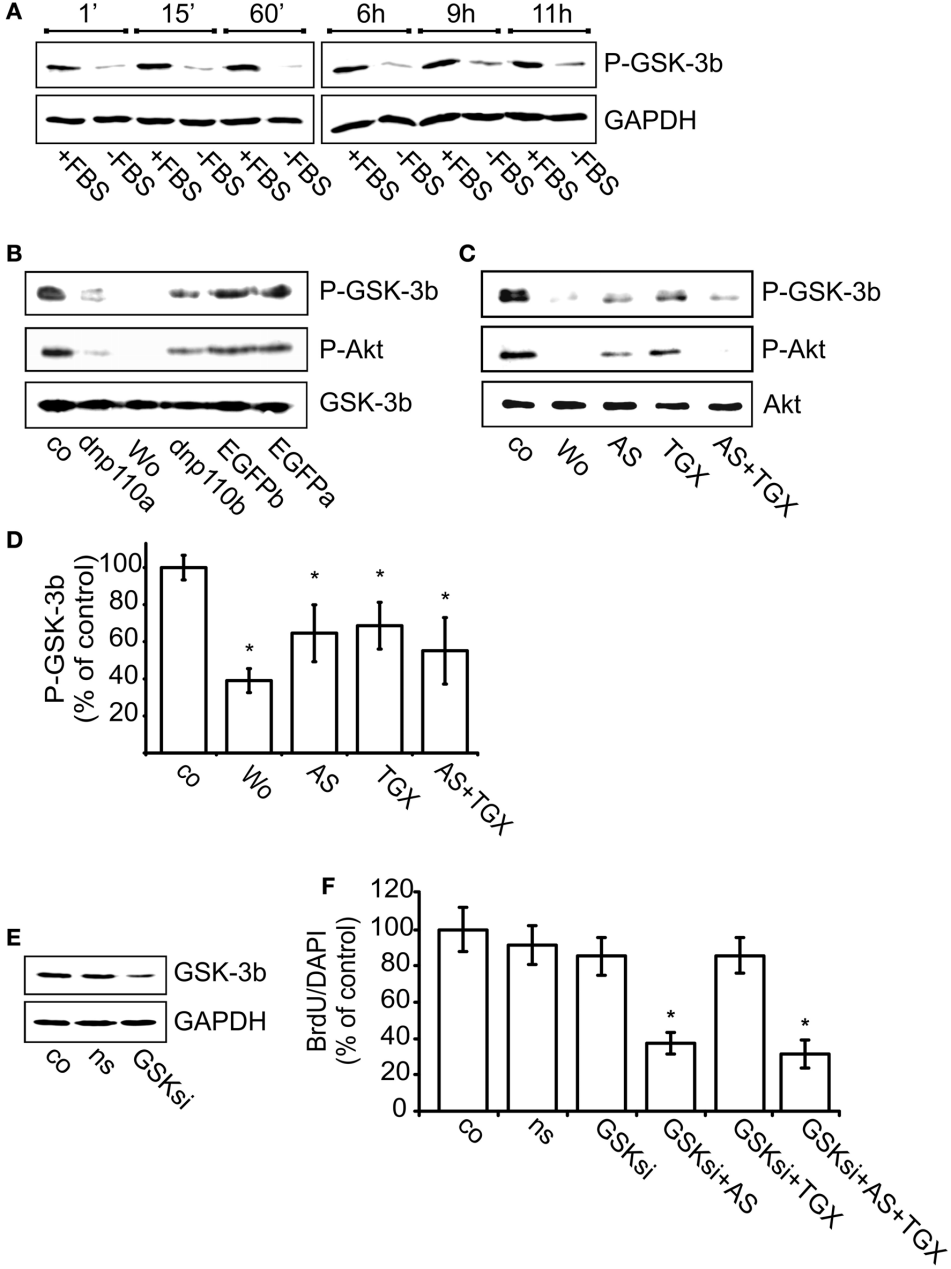
**Inhibition of p110β decreases the phosphorylation of GSK-3β and thereby reduces BrdU uptake. (A)** Upper lane: time-course of GSK-3β phosphorylation (P-GSK-3β) after FBS removal for 24 + n h (–FBS) as well as after re-addition of FBS for n h (+FBS). GAPDH served as input control (lower lane). **(B)** Upper lane: effects of dnp110α^K802R^ (dnp110α) and p110β^K805R^ (dnp110β) on the phosphorylation of GSK-3β. After prior FBS withdrawal, 10% FBS was added for 8 h. Vehicle-treated controls (co); controls for dnp110α^K802R^ ~ EGFPα; controls for p110β^K805R^ ~ EGFPβ Wortmannin (100 nM, wo) was added together with FBS. Middle lane: P-Akt in lysates of the same cells. Lower lane: total GSK-3β served as input control. **(C)** Upper lane: effects of AS-252424 (5 μM, AS) and TGX-221 (200 nM, TGX) on phosphorylation of GSK-3β. After prior FBS withdrawal, 10% FBS was added for 8h. Drugs were added together with FBS. **(D)** Quantification of Western blot experiments [mean ± CI (0.95) are shown; *n* = 4; ^*^~ *p* < 0.05 compared to controls]. **(E)** Upper lane: levels of GSK-3β protein in cells transfected for 48 h with GSK-3β siRNA (GSKsi) or non-silencing RNA (ns). GAPDH served as input control. **(F)** Effects of TGX-221 (200 nM, TGX) and AS-252424 (5 μM, AS) on nuclear uptake of BrdU in cells transfected with GSK-3β siRNA. For further explanation see Figures 2B,C. Mean ± CI (0.95) are shown; *n* ≥ 30; ^*^~ *p* < 0.05 compared to GSKsi group.

PI3-kinases inactivate GSK-3β by phosphorylating it at Ser9. Also in neocortical cultures, FBS withdrawal strongly reduced GSK-3β phosphorylation, whereas re-addition of FBS increased it (Figure 5A). Within 1 min after re-addition, FBS induced the phosphorylation of GSK-3β and maintained it during the next 11 h, the longest time-point measured. The subsequent experiment was performed 8 h after re-addition of FBS. Compared to the respective controls, expression of dnp110α^K802R^ or dnp110β^K805R^ significantly decreased the phosphorylation of GSK-3β (Figure 5B). Also pharmacological inhibition of p110α and p110β with AS-252424 or TGX-221 reduced the phosphorylation of GSK-3β by 40% (Figures 5C,D). Their combination reduced the cytosolic levels of P-GSK-3β and P-Akt like wortmannin. The latter reduced the phosphorylation of GSK-3β stronger than AS-252424 or TGX-221 given alone (Figures 5C,D).

Transfecting the cells with GSK-3β siRNA strongly reduced GSK-3β synthesis as compared to controls or cells transfected with non-silencing RNA (Figure 5E). In such cells RNA the number of nuclei labeled with BrdU remained unchanged as compared to controls (Figure 5F). In cells transfected with GSK-3β siRNA, however, the p110β inhibitor TGX-221 no longer reduced the number of BrdU labeled nuclei, whereas the p110α inhibitor AS-252424 reduced it by approximately 60% (Figure 5F). These data suggested that only p110β facilitated G1 phase progression by inhibiting GSK-3β.

### p110α and p110β facilitate nuclear BrdU uptake in the marginal zone of rat neocortex

**Figure 6:**
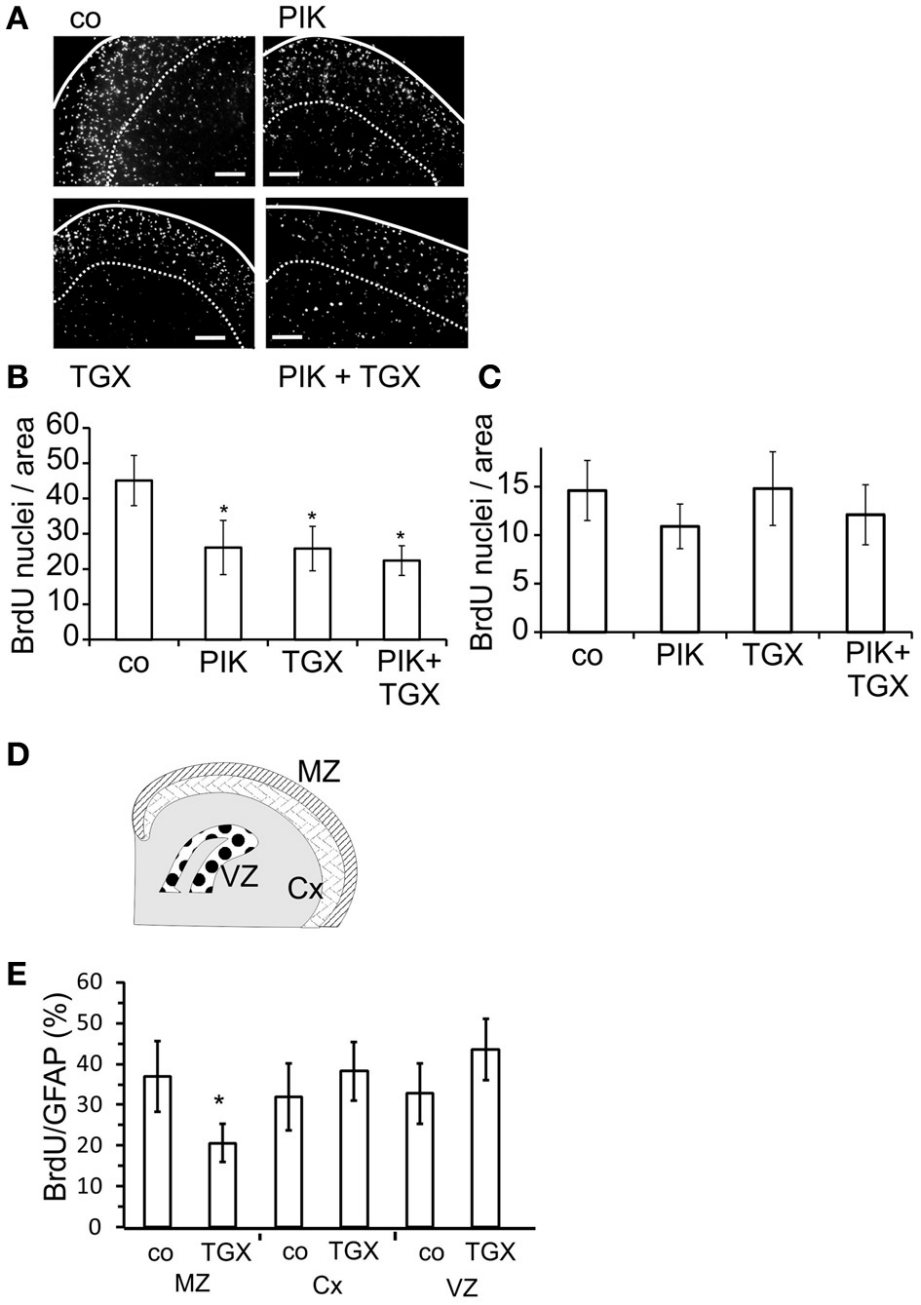
**Pharmacological inhibitors of p110α and p110β decrease the nuclear staining of BrdU in the marginal zone of the neocortex.** After 3 days in culture, slice cultures of the neocortex were treated for 4 days with 200 nM TGX-221 and/or 200 nM PIK-75, an inhibitor of p110α. **(A)** Micrographs of slices show BrdU stainings. Solid lines indicate pial surface; broken lines show the ventral border of marginal zone; calibration bars ~ 200 μm. **(B)** Quantitation of BrdU positive cells in the marginal zone. **(C)** Quantitation of BrdU positive cells in ventral layers of the neocortex. Means ± CI (0.95) are shown; *n* ≥ 17; ^*^~ *p* < 0.05 compared to controls. **(D)** Cartoon of the areas prepared shows marginal zone (MZ), deeper layers of neocortex (Cx), and ventricular zone (VZ). **(E)** Quantitation of nuclear staining of BrdU, which was compared to the number of GFAP positive cells. Means ± CI (0.95) are shown; *n* ≥ 8; ^*^~ *p* < 0.05 compared to controls.

Since our initial experiments suggested that PI3-kinases affected cell cycle progression only in a subgroup of neocortical astroglial cells, we studied whether these cells had a specific location. In organotypic slices prepared from neocortices of newborn rats and treated with BrdU from DIV 3–7, we analyzed the distribution of BrdU labeled nuclei. Close to the pial surface, labeled nuclei were more numerous than in deeper cortical areas (Figure 6A), confirming a previous observation by Ichikawa et al. (1983). When we applied the p110α inhibitor PIK-75 (200 nM) (Knight et al., 2006) or the p110β inhibitor TGX-221 (200 nM) from DIV 3–7, the number of BrdU labeled nuclei was reduced by approximately 50% in the area close to the pial surface, which corresponded to the MZ (Figure 6B). Combined application of both agents did not produce a stronger effect (Figure 6B). Applied alone or together, PIK-75 and TGX-221 did not affect the BrdU labeling in more ventral areas (Figure 6C).

To further investigate the area-dependent effects of the p110 inhibitors, we dissected neocortices from 5 days old rats into the MZ and deeper cortical layers (Cx) as well as the ventricular zone (VZ) (Figure 6D). In the resulting cultures, most cells were GFAP positive and thus considered to be astrocytes (data not shown). TGX-221 reduced the nuclear uptake of BrdU only in astroglial cells prepared from the MZ of the neocortex (Figure 6E).

## Discussion

In cultured neocortical astroglial cells, p110α and p110β mediated nuclear uptake of BrdU via different mechanisms. Whereas p110α reduced the protein levels of the CDK2-inhibitor p27^Kip1^, p110β phosphorylated and inactivated GSK-3β. The PI3-kinases facilitated S phase entry only in cells dissected from the MZ of the neocortex.

Neocortical astroglial cells are generated during sequential stages of development. During embryonic development, radial glial cells in the VZ are transformed into astroglial cells (Choi and Lapham, 1978; Schmechel and Rakic, 1979; Kriegstein and Alvarez-Buylla, 2009). During the early postnatal period, intermediate progenitor cells originating from radial glial and/or neuroepithelial cells proliferate and produce astroglial cells. The radial glial cells and progenitors are located in the ventricular and subventricular zone (Grove et al., 1993; Luskin et al., 1993; Qian et al., 2000; McCarthy et al., 2001; Kriegstein and Alvarez-Buylla, 2009). During embryonic development, additional neural progenitors appear in the preplate and MZ. They continue to proliferate during the peripartal period and give rise to neurons, astroglial, and oligodendroglial cells which are mainly located in the outer Cx (Costa et al., 2007). Thus, astroglial cells from distinct neocortical areas differ in their origin. The present data show that PI3-kinases regulate S phase entry in a subgroup of astroglial cells. Only in cells dissected from the MZ, inhibition of PI3-kinases reduced by approximately 40% the number of BrdU labeled nuclei. Combined application of p110α and p110β inhibitors caused a stronger reduction in nuclear BrdU uptake than inhibition of a single isoform. Whether both isoforms were expressed in two distinct cell populations remained unclear. In most neocortical astroglial cells, S phase entry was independent of endogenous PI3-kinases. This finding is in agreement with data that genetic inactivation of p110β does not induce striking changes in cortical development (Ciraolo et al., 2008). *In vivo*, astroglial cells which enter S phase independently of PI3-kinases may compensate for the lack of PI3-kinase dependent cells. After 10 DIV, PI3-kinase inhibition reduced the number of BrdU labeled nuclei by approximately 50% in our cultures, suggesting a rapid growth of this subgroup.

Cultured astroglial cells harvested from deep neocortical layers also showed nuclear BrdU uptake, which was independent of PI3-kinases. Some of these deep layer cells may have been be silent astrocytes, which regained the ability to proliferate in response to the dissociation procedure (Buffo et al., 2008).

According to genetic evidence, p110β may have functions independent of its kinase activity. Homozygous mice with deleted p110α or p110β die during early embryonic development (Bi et al., 1999, 2002). Also knock-in mice expressing kinase-dead p110α die at an early embryonic stage (Foukas et al., 2006), whereas knock-in mice expressing kinase-dead p110β appear to be normal (Ciraolo et al., 2008). Moreover, deletion of p110β reduces the proliferation of mouse embryonic fibroblasts, whereas proliferation is unaltered in fibroblasts expressing kinase-dead p110β. Consequently, it has been suggested that non-catalytic p110β may act as a scaffold for proteins involved in proliferation (Ciraolo et al., 2008; Jia et al., 2008). In our experiments, the kinase-dead isoform p110β^K805R^ as well as TGX-221, which blocks the ATP binding site of p110β (Condliffe et al., 2005; Knight et al., 2006) reduced nuclear BrdU uptake, indicating that the kinase domain was involved.

PI3-kinases are known to regulate the cellular levels of p27^Kip1^, which inhibits the cyclin E/CDK2 complex (Bacqueville et al., 1998; Collado et al., 2000; Graff et al., 2000; van Duijn and Trapman, 2006; Chu et al., 2008; Vervoorts and Luscher, 2008). Dnp110α^K802R^ and the p110α inhibitor AS-252424 enhanced the cellular level of p27^Kip1^ in our cultures, whereas dnp110β^K805R^ and the p110β inhibitor TGX-221 were without effect. This increase in p27^Kip1^ levels was linked to BrdU labeling. Transfection of the cells with p27^Kip1^siRNA reduced the cellular level of p27^Kip1^, enhanced the number of BrdU labeled nuclei and indeed prevented the reducing effect of AS-252424. In contrast, in cells treated with p27^Kip1^siRNA the effect of TGX-221 on nuclear BrdU labeling remained unchanged. Taken together, these data strongly suggest that p110α mediates the G1/S phase transition by lowering p27^Kip1^ levels. Synthesis and degradation tightly regulate the cellular levels of p27^Kip1^. Members of the class O of forkhead transcription factors (FOXOs) enhance the cellular level of p27^Kip1^ (Medema et al., 2000; Stahl et al., 2002). PI3-kinases phosphorylate FOXOs. They also phosphorylate p27^Kip1^ and thus cause its cytosolic accumulation and inactivation (Marques et al., 2008). Moreover, PI3-kinases can induce the ubiquitination and proteasomal degradation of p27^Kip1^ (Nakayama and Nakayama, 2006). Which of these actions was exerted by p110α in our astroglial cells remained open.

In our cultures, both p110α and p110β phosphorylated GSK-3β at Ser9 confirming previous data (Cross et al., 1995). However, in cells treated with GSK-3β siRNA or the antagonist CHIR-99021 only the p110β antagonist TGX-221 lost its inhibitory effect on nuclear labeling with BrdU, whereas the p110α inhibitor AS-252424 remained active. Apparently, only p110β facilitated nuclear BrdU labeling by inhibiting GSK-3β GSK-3β can reduce proliferation by initiating the translocation of cyclin D from the nucleus to the cytosol and thus its ubiquitin-dependent proteolysis. This process can be blocked by PI3-kinases (Diehl et al., 1998). Inhibition of GSK-3β also stabilizes β-catenin in its effect on proliferation in neuronal progenitors (Mao et al., 2009).

Infection of the cells with an adenoviral vector coding for wtp110α increased by 120% the number of BrdU labeled nuclei as compared to controls. In glioblastoma multiforme, loss-of-function mutations of the phosphatase PTEN which enhance PI(3,4,5)P_3_ levels as well as gain of function mutations of p110α and of the regulatory subunit p85 have been found. Therefore, it has been suggested that PI3-kinases may be involved in the pathogenesis of such tumors (Ligresti et al., 2009; Jones and Holland, 2011a,b). In glioma cell lines, inhibition of GSK-3β by LiCl indeed increases proliferation (Atkins et al., 2012). Whether the tumorigenic contribution of PI3-kinases depends on the location and origin of the glial cells, has to be further studied.

### Conflict of interest statement

The authors declare that the research was conducted in the absence of any commercial or financial relationships that could be construed as a potential conflict of interest.
